# Domestic Pigs and Japanese Encephalitis Virus Infection, Australia

**DOI:** 10.3201/eid1411.071368

**Published:** 2008-11

**Authors:** Andrew F. van den Hurk, Scott A. Ritchie, Cheryl A. Johansen, John S. Mackenzie, Greg A. Smith

**Affiliations:** Queensland Health, Coopers Plains, Queensland, Australia (A.F. van den Hurk, G.A. Smith); The University of Queensland, St. Lucia, Queensland, Australia (A.F. van den Hurk); Queensland Health, Cairns, Queensland, Australia (S.A. Ritchie); James Cook University, Cairns (S.A. Ritchie); The University of Western Australia, Nedlands, Western Australia, Australia (C.A. Johansen); Australian Biosecurity Cooperative Research Centre for Emerging infectious Diseases, Perth, Western Australia, Australia (J.S. Mackenzie)

**Keywords:** Japanese encephalitis virus, domestic pigs, mosquito infection, Australia, dispatch

## Abstract

To determine whether relocating domestic pigs, the amplifying host of Japanese encephalitis virus (JEV), decreased the risk for JEV transmission to humans in northern Australia, we collected mosquitoes for virus detection. Detection of JEV in mosquitoes after pig relocation indicates that pig relocation did not eliminate JEV risk.

Japanese encephalitis virus (JEV) is a major cause of viral encephalitis in Southeast Asia; >50,000 cases are reported annually (*1*). Ardeid wading birds are the primary maintenance hosts, pigs are the main amplifying hosts, and *Culex* mosquitoes are the primary mosquito vectors. Suppression of JEV disease in humans is generally considered to be best achieved through vaccination of humans or swine, mosquito control, or a combination of these strategies (*2*). An alternative approach of moving domestic pigs away from human habitation has been suggested as a potential method of reducing JEV transmission to humans (*1*,*3*). Although this strategy could be considered a logical way to limit human exposure to infected vectors, the actual effect that removing domestic pigs would have on mosquito infection rates has not been established.

Since the emergence of JEV in northern Australia in 1995, we (the authors) have investigated the ecology of JEV on Badu Island in the Torres Strait, where most human, pig, and animal infections have occurred. Intense transmission on this island has been linked to domestic pigs, which until late 1998 were housed in small backyard pens (Figure 1, panel **A**), and high populations of *Culex sitiens* subgroup mosquitoes (*4*). In Australia, members of the *Cx*. *sitiens* subgroup, from which *Cx*. *annulirostris* is considered to be the most important species, are the primary Australian JEV vectors (*5*). During an outbreak in 1998, the virus was shown to be widespread on Badu Island; isolates were obtained from mosquitoes collected throughout the community (*6*). A vaccination program initiated on the outer Torres Strait islands in 1995, including Badu Island, appears to have limited the number of human clinical cases (*5*).

**Figure 1:**
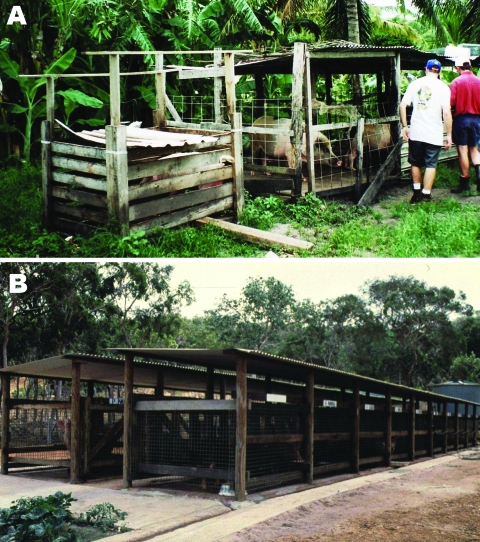
Pig housing in Badu Island. A) Typical backyard pig pen in community before removal in 1998 and B) Badu Island piggery, where pigs have been housed since late 1998.

To further reduce JEV risk for Badu Island residents, after the 1998 outbreak domestic pigs were removed from the Badu Island community to a piggery located ≈2.5 km away (Figure 1, panel **B**). This relocation led to a significant reduction in the proportion of *Cx*. *annulirostris* feeding on pigs, and speculation was that this might reduce the number of JEV-infected mosquitoes (*7*). We report on the effects of pig relocation away from human habitation on virus infection rate in *Cx*. *sitiens* subgroup mosquitoes.

## The Study

In response to JEV activity, as evidenced by human clinical cases or the seroconversion of sentinel pigs, adult mosquitoes were collected on Badu Island during 1995, 1998, and 2003 (*6,8,* and this article, respectively). Badu Island is located at 10º07′S and 142º09′E in the central western region of the Torres Strait and is a granite island of ≈101 km^2^; its ecology has been described (*6*–*8*).

The mosquitoes were collected with Centers for Disease Control (CDC) miniature light traps (Model 512; John W. Hock Co., Gainesville, FL, USA) baited with either CO_2_ alone or in combination with 1-octen-3-ol. Mosquitoes were killed on dry ice and placed in liquid nitrogen dry shippers or on dry ice in insulated containers for transport to Cairns for storage at –70^o^C. Mosquitoes were placed on a refrigerated table for species or taxonomic group identification before being placed in pools of <200 mosquitoes and sent to Queensland Health Forensic and Scientific Services or the University of Queensland, Brisbane, for JEV detection.

The virus isolation protocols used in 1995 and 1998 have been described (*6*,*8*). In 2003, virus RNA was detected by using a real-time TaqMan reverse transcription–PCR (*9*).

**Figure 2:**
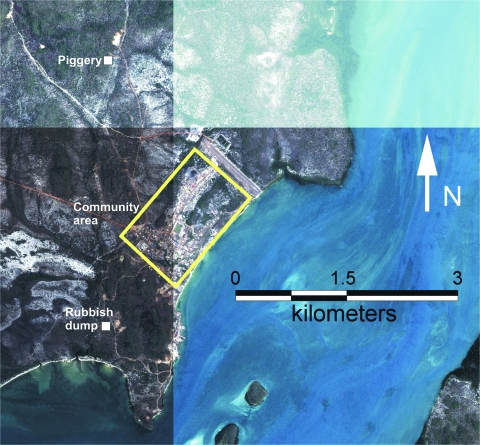
Aerial photograph of Badu Island showing the location of the community, piggery, and rubbish dump.

To facilitate the comparison of virus distribution on Badu Island, trap locations were grouped into 3 general areas: within 1.2 km of the piggery, within the area of human habitation (the community), and at a rubbish dump located ≈1.5 km from the community and 4.0 km from the piggery (Figure 2). Dumps are a potential focus of JEV activity because mosquitoes, feral pigs, and wading birds congregate at them (*10*). Maximum-likelihood estimation of mosquito infection rates with 95% confidence intervals were calculated for each of these general areas by using the PooledInfRate statistical software package (*11*).

Because of the difficulty in morphologically separating the members of the *Cx*. *sitiens* subgroup, data for the 3 members of the group found in Australia—*Cx*. *annulirostris*, *Cx*. *palpalis*, and *Cx*. *sitiens—*were pooled for analysis. However, PCR restriction fragment length polymorphism analysis of a subsample of 135 *Cx*. *sitiens* subgroup mosquitoes collected in 2003 showed that *Cx*. *annulirostris* was the dominant member of this group on Badu Island and comprised 94.1% of polymorphic specimens processed; the other 4.4% and 1.5% were identified as *Cx*. *palpalis* and *Cx*. *sitiens*, respectively.

A total of 44,328 *Cx*. *sitiens* subgroup mosquitoes were processed for detection of JEV; 2,871, 24,592, and 16,865 were processed from 1995, 1998, and 2003, respectively (Table). JEV was detected in 66 pools of *Cx*. *sitiens* subgroup mosquitoes; the highest maximum-likelihood estimation of mosquito infection rate was obtained from mosquitoes collected at the dump in 1998. Despite removal of the domestic pigs, JEV was still detected in 5 pools of *Cx*. *sitiens* subgroup mosquitoes collected within the community in 2003. However, the point estimates of infection rates were lower than those obtained in 1995 and 1998, when domestic pigs were present within the community, although the slight overlap in 95% confidence intervals indicates that this difference in infection rate was not significant.

**Table:** Mosquito infection rates during 3 recognized incursions of Japanese encephalitis virus, Badu Island, northern Australia*

Collection location	Pigs located within community								Pigs relocated outside community		
	1995				1998				2003		
	No.†	No. detected‡	Infection rate (95% CI)		No.†	No. detected‡	Infection rate (95% CI)		No.†	No. detected‡	Infection rate (95% CI)
Community	2,871	8	3.02 (1.43–5.74)		23,467	38	1.69 (1.21–2.29)		7,019	5	0.75 (0.28–1.66)
Piggery	NS	NS	NS		NS	NS	NS		3,316	5	1.61 (0.61–3.56)
Dump	NS	NS	NS		1,125	4	3.68 (1.20–8.85)		6,530	6	0.99 (0.41–2.07)

*Mosquito infection rates determined by maximum-likelihood estimation; 1995, Apr 8–9 and 20–21, 30 trap nights; 1998, Mar 5–6, 25 trap nights; 2003, Mar 13–19, 92 trap nights; CI, confidence interval; NS, mosquitoes not sampled from this location during the year of collection. †Total no. mosquitoes processed. ‡No. Japanese encephalitis virus–positive pools detected by virus isolation or TaqMan reverse transcription–PCR.

## Conclusions

We demonstrated that although removing domestic pigs from areas of human habitation may reduce contact between amplifying hosts and vectors (*7*), it does not eliminate the presence of JEV-infected mosquitoes. Thus, pig removal does not negate JEV risk for humans. Indeed, evidence for low-level virus transmission to humans is provided by Hanna et al., who found a low level (32%) of natural boosting immunity in Badu residents who had received an inactivated mouse brain–derived JEV vaccine 3 years earlier (*12*).

As has been observed elsewhere in the absence of pigs (*13*), mosquitoes may have become infected by feeding on viremic herons and egrets, populations of which are found on Badu Island (*14*). Analysis of host feeding patterns demonstrated that birds accounted for 23% of blood meals of *Cx*. *annulirostris* identified from the dump in 2003 (S. Hall-Mendelin and A.F. van den Hurk, unpub. data). Alternately, feral pigs in the community and at the dump are a potential source of virus for mosquitoes, although <1% of mosquito blood meals were from swine at these locations.

Mosquitoes could become infected by feeding on viremic pigs at the piggery and then disperse to other areas on the island. The mean flight distance of *Cx*. *annulirostris* is 4.4 km; some females traverse up to 12 km (*15*), which is considerably farther than the 2.5 km between the piggery and the community on Badu Island. Solomon recommends that domestic pigs be moved >5 km from human habitation to limit JEV transmission to humans (*3*). Indeed, if infected mosquitoes were flying from the piggery to the community, then the data from our study support this recommendation. However, we suggest that domestic pigs be removed far enough away from human habitation to encompass the flight range of the local *Culex* vectors.
